# Pharmacists’ perspectives and attitudes towards the 2021 down-scheduling of melatonin in Australia using the Theoretical Domains Framework: a mixed-methods study

**DOI:** 10.1007/s11096-023-01605-w

**Published:** 2023-06-24

**Authors:** Kingston W. C. M. Yeung, Samantha K. M. Lee, Yu Sun Bin, Janet M. Y. Cheung

**Affiliations:** 1 Sydney Pharmacy School, Faculty of Medicine and Health, The University of Sydney, Pharmacy and Bank Building (A15), Science Road, Camperdown Campus, Sydney, NSW 2006 Australia; 2 Sleep and Circadian Research Group, Woolcock Institute of Medical Research, 431 Glebe Point Road, Glebe, Sydney, NSW 2037 Australia; 3 Northern Clinical School, Sydney Medical School, Faculty of Medicine and Health, The University of Sydney, Sydney, NSW 2006 Australia; 4 Sleep Research Group, Charles Perkins Centre, The University of Sydney, Sydney, NSW 2006 Australia

**Keywords:** Community pharmacy, Continuing pharmacy education, Insomnia, Melatonin, Pharmaceutical scheduling, Pharmacy legislation, Pharmacy practice, Qualitative research

## Abstract

**Background:**

In Australia, prescription melatonin became a ‘Pharmacist Only Medicine’ for people over 55 with insomnia from June 2021. However, little is known about pharmacists’ views on melatonin down-scheduling and perceived impacts on practice.

**Aim:**

To explore Australian community pharmacists’ views on and attitudes towards the down-scheduling of melatonin.

**Method:**

A convenience sample of community pharmacists and pharmacy interns were recruited. Participants completed a survey capturing demographic and professional practice details, and rated their knowledge, beliefs and attitudes towards melatonin. This was followed by an online semi-structured interview. Interviews were guided by a schedule of questions developed using the Theoretical Domains Framework and explored the perceived role of melatonin, preparation/response to down-scheduling, practice changes and patient interactions. Interviews continued until data saturation and were digitally recorded, transcribed verbatim and analysed using the Framework Approach.

**Results:**

Twenty-four interviews were conducted with community pharmacists (n = 19) and intern pharmacists (n = 5), all practicing in metropolitan areas. Pharmacists/intern pharmacists welcomed the increased accessibility of melatonin for patients. However, pharmacists perceived a disconnect between the guidelines, supply protocols and pack sizes with practice, making it difficult to monitor patient use of melatonin. The miscommunication of eligibility also contributed to patient-pharmacist tension when supply was denied. Importantly, most participants indicated their interest in upskilling their knowledge in melatonin use in sleep, specifically formulation differences and dosage titration.

**Conclusion:**

While pharmacists welcomed the down-scheduling of melatonin, several challenges were noted, contributing to pharmacist-patient tensions in practice. Findings highlight the need to refine and unify melatonin supply protocols and amend pack sizes to reflect guideline recommendations as well as better educating the public about the risk-benefits of melatonin.

**Supplementary Information:**

The online version contains supplementary material available at 10.1007/s11096-023-01605-w.

## Impact statements

- Refining and unifying melatonin supply protocols and amending pack sizes to reflect treatment duration is needed to support pharmacists’/interns’ provision of melatonin to criteria-concordant patients.
- Public education about the risk-benefit of melatonin use is needed to facilitate appropriate use of melatonin and to diffuse patient-pharmacist tension when supply is denied.
- Further continuing professional development and training for pharmacists in sleep and circadian health is needed.

## Introduction

Insomnia is one of the most prevalent presenting complaints in community pharmacy, [1] with up to 60% of the Australian population experiencing at least one symptom (e.g., difficulty falling asleep, staying asleep) despite adequate sleep opportunity [2]. Clinically, insomnia assumes a disorder status when patients experience daytime deficits resulting from poor sleep [3]. The aetiology of insomnia is multifactorial with psychophysiological factors. The 3-P model attributes insomnia to various predisposing (e.g., personality traits), precipitating (e.g., stressful life event) and perpetuating factors (e.g., learned behaviours and beliefs that negatively affect sleep ability) [4]. The latter plays a role in maintaining maladaptive sleep beliefs/behaviours that contributes to the development of chronic insomnia [5]. Currently, cognitive behavioural therapy for insomnia (CBT-I) is considered the gold standard treatment for insomnia. Where CBT-I is not effective or unavailable, pharmacotherapy (e.g., benzodiazepines receptor agonists and orexin receptor antagonists) is recommended for short-term use of 2–3 weeks [6, 7]. Despite the availability of evidence-based treatments, there is a strong propensity among individuals experiencing insomnia to defer medical care in favour of self-management/self-treatment [8–10]. Consequently, natural products (NPs) and over-the-counter (OTC) sleep aids such as sedating antihistamines are commonly sought by individuals with insomnia [11]. As healthcare providers who are highly accessible to the public, community pharmacists play a central role in promoting quality use of medicines, patient counselling, and further referral.

Prolonged-release (PR) melatonin containing 2 mg or less has recently been added to Australian pharmacists’ repertoire of sleep aids, alongside NPs (e.g., valerian) and other OTC options (e.g., doxylamine) [12]. Melatonin is a chronobiotic, whose effects are mediated through activity on Melatonin Type 1 and Melatonin Type 2 receptors [13]. Despite the widespread availability of melatonin as a dietary supplement in North America [14], PR-melatonin was only available by prescription in Australia until 1st June 2021. Under this regulatory change, PR-melatonin (2 mg or less) is now available OTC as a Schedule 3 (S3) Pharmacist-Only Medicine^1^ for those over the age of 55 with primary insomnia to address age-related decline in melatonin levels [12, 16]. The down-scheduling of melatonin resulted from a reassessment of risk-benefit to public access by the Therapeutic Goods Administration (TGA), the Australian government authority that regulates the quality and supply of medicines. The decision was based on melatonin being a safer alternative to current pharmacological treatment options in patients aged over 55 [12, 17]. The potential for abuse/harm is low from a pharmacovigilance standpoint, but the chronobiotic properties of melatonin make it amenable to inappropriate use. The standard recommendation to “take melatonin 1–2 hours before bedtime” [18], could potentially lead to inappropriate circadian phase shifting^2^, worsening the original sleep complaint. Ideally, the administration of melatonin is in alignment with the individual’s circadian rhythmicity as determined by their dim light melatonin onset (DLMO), which typically occurs about 2 h before habitual bedtime [20]. Taking melatonin too early in the evening may unintentionally phase-advance^3^ the older individual depending on their existing circadian rhythmicity [21]. However, principles of chronotherapy are rarely considered in practice. Much like the up-scheduling of codeine-containing products in 2018, pharmacists will likely need further practice support and education in the evolving area of melatonin provision and point-of-care patient management [22, 23]. However, little is known pharmacists’ views on melatonin down-scheduling or their perceived impacts on practice.

### Aim

To explore Australian community pharmacists’ experiences, views, and attitudes towards the down-scheduling of melatonin. Specifically, we sought to gain insight into how pharmacists’ have responded to and adapted their practice.

### Ethics approval

All research materials and protocols were approved by the University of Sydney Human Research Ethics Committee (HREC: [2022/210]). Prior to commencing, participants were provided with information outlining the study protocol and research dissemination plan. Participant consent was implied upon the submission of survey responses and verbally verified again prior to the commencement of each interview. Only aggregated/de-identified data are presented.

## Method

### Study design and setting

The present study is part of a larger mixed methods study conducted by the research team exploring the current state of sleep health management by community pharmacists in Australia. The broader study covers issues relating to pharmacists’ current approaches in managing and referring patients for common sleep disorders, provision of melatonin and interacting with caregivers of children using melatonin for sleep. This paper will focus on exploring pharmacists’ experiences, views, and attitudes towards the down-scheduling of melatonin in community pharmacy for adults with insomnia aged 55 years and over in an Australian practice context. In North America, melatonin is regarded as a dietary supplement and does not require a prescription. While this can be regarded as OTC, there is no requirement for a pharmacist to be involved in the sale of the product [24]. In Australia, a pharmacist is required by legislation to be involved in assessing the appropriateness of melatonin for the presenting patient prior to supply [15]. In the United Kingdom melatonin is only available via prescription. Within Europe, the lack of harmonisation in the provision of melatonin provision leads to variability in its regulatory status (i.e., OTC vs. prescription) from country to country[25, 26], thus requiring varying levels of pharmacist involvement in its provision.

### Recruitment

All community pharmacists (~ 16,000) and intern pharmacists (~ 2,000) across Australia were eligible for inclusion in this study [27]. Recruitment initially focused on a convenience sample from metropolitan Sydney and metropolitan Wollongong, cities in the state of New South Wales (NSW) based on professional networks known to researchers. Further recruitment occurred through social media, and participants were asked to share and discuss the study within their professional networks. Interested colleagues were provided with the researchers’ contact details and the study link. Recruitment continued until data saturation was reached [28, 29]. This was denoted by three consecutive interviews with no additional content after the anticipated 20 interviews required to reach saturation.

### Data collection: survey

Prospective participants completed an online survey capturing their demographic information and details about their professional practice setting. Given the lack of validated instruments available for evaluating pharmacists’ knowledge and attitudes towards sleep health, ten additional items were developed in reference to the relevant adjacent literature [30, 31], to gauge participants’ knowledge, beliefs, and attitudes towards melatonin use for sleep. Beliefs and attitudes were captured as a 5-point Likert-scale (1 = strongly agree; 2 = agree, 3 = neither agree nor disagree, 4 = disagree 5 = strongly disagree). Participants’ counselling strategies during the provision of OTC PR-melatonin was captured as a binary outcome (i.e., yes/no). As part of the survey, participants indicated their interest in being interviewed. In this study, only the survey data from participants interviewed are presented (Online Resource 1).

### Data collection: semi-structured interviews

The semi-structured interviews sought to capture participants’ practice experiences in light of the recent down-scheduling of PR-melatonin. The interview guide was conceptualised by the senior author (female, registered pharmacist) in collaboration with a public health expert on the research team. Concepts from the Theoretical Domains Framework (TDF) were utilised to develop a schedule of interview questions to understand how pharmacists responded to the down-scheduling of melatonin [32]. TDF provides a theoretical lens to understand cognitive, affective, social, and environmental behavioural influences. It is widely used in implementation research to understand and facilitate behaviour change among healthcare professionals (e.g., uptake/ non-uptake of evidence-based behaviours) [33, 34]. Key topics explored in the interviews, framed around the 14 core domains of TDF, included pharmacists’ perceived appropriateness of present guidelines and training materials, pharmacists’ perceived preparedness for down-scheduling, and the impact of down-scheduling on practice (Online Resource 2). The included questions were largely informed by the literature on over-the-counter sleep aid provision in the Australian context [1, 35] along with the observed trends in the regulation of melatonin (i.e., down-scheduling and introducing new paediatric formulations for melatonin) and framed within the respective domains of the TDF by JC and SL. The interview guide was first piloted with two pharmacists and feedback was used to further refine the document, but these initial interviews were not included in the final analysis. Subsequent interviews were conducted with participants via Zoom by two researchers: KY (male, final year pharmacy student and pharmacy assistant in community pharmacy) and SL (female, registered pharmacist, and master’s graduate student). All interviews were digitally recorded and transcribed verbatim. Field notes were taken to facilitate analysis.

### Quantitative data analysis

Data collected from the surveys were descriptively analyzed using IBM SPSS Statistics version 26 [36].

### Qualitative data analysis

Recorded interviews were transcribed verbatim and cross-checked against the recordings for fidelity. The Framework Approach (FA) was used for data analysis, facilitated using the QSR NVivo software [37]. FA evolved from applied social policy research but is not aligned with a particular epistemological approach, allowing for flexibility to analyse data through both an inductive and deductive lens [38, 39]. FA involves five major steps: familiarisation, developing a thematic framework, indexing, charting and mapping, and interpretation [39].

FA starts with *familiarising* data through iteratively reading interview transcripts and field notes to gain insight into emerging ideas and concepts that participants have generated. The next step involves *identifying a thematic framework*, where emergent concepts from participant interviews were integrated with *a priori* research questions that we sought to explore at the study’s outset. The thematic framework was developed by all members of the research team. A preliminary thematic framework was developed and piloted against a random selection of 6 interviews and subsequently refined by JC, KY and SL. During *indexing*, the thematic framework is systematically applied to each interview transcript, identifying and coding relevant sections. The fourth step, *charting*, involves identifying relationships between various thematic categories to rearrange data into thematic matrices to form chart summaries. In the final stage, *mapping and interpretation*, within-case and cross-case associations are explored to describe the phenomena of interest and identify emergent themes. Three members of the research team (KY, JC, and SL) coded the interviews and abstracted codes into emergent themes. Coding trees representing the codes, categories, and emergent themes are provided in Online Resource 3a-3c. A subset of interviews (n = 10) was randomly selected to cross-check for inter-coder reliability by KY, JC, and SL to ensure the reliability of thematic categories against the framework (Kappa Score = 0.89). Any disagreement on thematic categories was resolved through discussion. Where a consensus cannot be reached, the fourth researcher (YSB) was consulted. The study adhered to the Consolidated Criteria for Reporting Qualitative Research (COREQ) checklist [40] (Online Resource 4).

## Results

### Participant characteristics

Out of 103 survey completers, 24 agreed to be interviewed (participation rate = 23%). Twenty-four interviews were conducted with pharmacists (n = 19) and intern pharmacists (n = 5) practising in Australian community pharmacies between July and September 2022. The mean duration of the interview was 26.4 min (SD = 6.5, Range = 18 to 49 min). Participants had a mean age of 30 years (SD = 9.1, Range = 22 to 51) and were primarily female (67%). All interviewees practiced in metropolitan areas and the majority were registered pharmacists (n = 15) working in franchise pharmacies (n = 14).

### Quantitative survey results

Pharmacists reported dealing with an average of 16 sleep complaints per day (SD = 29.3, Range = 3 to 150). Most pharmacists (n = 17) considered melatonin a hormonal agent, and half (n = 12) had not undergone targeted training before the down-scheduling. All participants reported increased melatonin inquiries, while the sale of other S3 sleep aids remained unchanged for 80% of participants (n = 20) (Table 1).

**Table 1:** Participant characteristics

Variable		Participant (n = 24) % (n) or Mean (SD)
Gender	Male	33% (8)
	Female	67% (16)
Mean Age		30.0 (9.1)
Mean Years in Practice		7.3 (9.5)
Role in Pharmacy	Intern pharmacist	21% (5)
	Registered pharmacist	63% (15)
	Pharmacist in charge	13% (3)
	Pharmacist manager	4% (1)
Type of Practice	Independent or private pharmacy	38% (9)
	Franchise pharmacy	58% (14)
	GP clinic pharmacy	4% (1)
Average estimated number of prescriptions dispensed per day		365.4 (162.5)
Mean estimated number of patients presenting with sleep complaints per day		15.7 (29.3)
Mean estimated requests for melatonin per week		18.3 (17.1)
Perceived main function of melatonin in sleep	Sleep-inducing agent	29% (7)
	Hormonal agent	71% (17)
	Chronobiotic agent	42% (10)
Training accessed prior to down-scheduling*	Pharmaceutical society of Australia (PSA)	33% (8)
	Australian Journal of Pharmacy (AJP)	33% (8)
	Other	4% (1)
	No training	50% (12)
Compounding services for melatonin offered	Yes	21% (5)
Perceived sales of other S3 sleep aids since the down-scheduling of melatonin	Increased	54% (3)
	Decreased	4% (1)
	Stayed the same	83% (20)
Record supply of S3 Melatonin in Pharmacy	Yes	54% (13)
Perceived workload changes since the down-scheduling of melatonin.	Increased more than usual	46% (11)
	Remains unchanged	54% (13)
Mean interview duration in minutes		26.4 (6.5)

*Numbers do not add to 100% as participants can choose > 1 option in their response

**Fig. 1:**
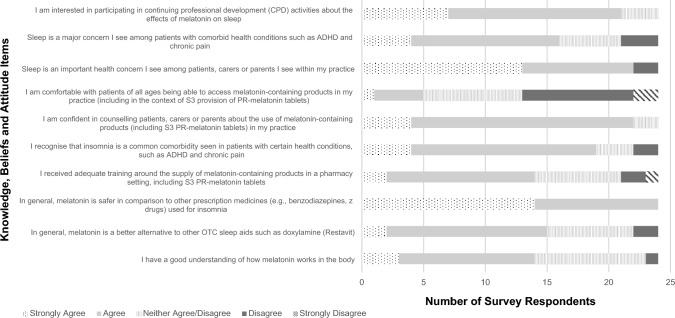
Participant knowledge, beliefs and attitudes towards melatonin use and sleep

**Fig. 2:**
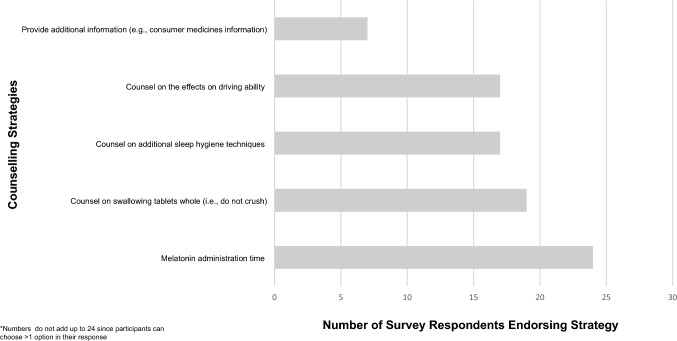
Frequency of pharmacist counselling strategies in relation to melatonin use

From Figs. 1 and 2, pharmacists/intern pharmacists endorsed sleep health as a frequent concern in practice with 19 agreeing that sleep is an important concern among patients with comorbid conditions. Interestingly, all participants agreed that melatonin is safer than prescription sleep aids, but 9 of pharmacists/intern pharmacists were either impartial or did not agree that melatonin was better than existing OTC sleep aids. With respect to the provision of melatonin, 22 participants agree/strongly agree that they were confident in counselling patients in the use of melatonin, but only 5 were comfortable with widening melatonin use to all age groups. Interest in undertaking further continuing professional activities to learn about the effects of melatonin on sleep was endorsed by 21 participants. With respect to counselling, all 24 participants endorsed counselling on the timing of melatonin administration. Advice against crushing the tablet was the next most frequently endorsed (n = 19), followed by sleep hygiene techniques and warnings about impaired concentration/driving (n = 17). Only 7 endorsed providing patient information on melatonin (Fig. 2).

### Qualitative interview results

Analysis of qualitative data revealed 3 key emergent themes. Tables 2, 3 and 4 provides a summary of the thematic synthesis and illustrative quotes for emergent themes.

**Table 2:** Summary of qualitative thematic synthesis for Theme 1

Theme 1: Role of melatonin: beliefs and attitudes		
Subthemes	Summary of synthesis	Illustrative participant quotes ^‡^
Subtheme 1.1 Role of melatonin: regulating rather than inducing sleep	• Melatonin role in circadian rhythm for regulating sleep-wake cycles. • Exogenous melatonin supplementation for replenishing declining levels in older patients. • Melatonin gradually recalibrates sleep patterns - more important role for chronic insomnia compared to acute insomnia.	• “Sort of makes your body ready for sleep, and when you’ve got higher levels of that, it’s sleeping time. And when you’ve got lower levels, it’s waking time.”**(P18, 45 M)** • “… those who are over a certain age, especially in the elderly population, they tend to have lower melatonin as they grow older. So it’s really nice to have melatonin to help them just have a little boost of melatonin at night time, so they can at least get a boost to get them to sleep…”**(P15, 23 F)** • “So, if there’s known cause for sleeping problems, shift work for example, then maybe all they need is a short-term sedative, Restavit™^†^, just to help them sleep. Just to help them get back into normal sleeping routine. Whereas if it’s more of a long term thing, then maybe try Circadin^*^will be more effective, because their sleeping, if their sleeping cycle has been out of whack for a very long time, you probably need something on a longer period of time to help them get back into that rhythm.” **(P17, 30 M)**
Subtheme 1.2 Comparing sleep aids	• Melatonin perceived as natural and therefore possesses a wider safety window compared to prescription sleep aids with respect to abuse potential, tolerance and side effects. • When compared to natural products (e.g., valerian), pharmacists felt the body of evidence for melatonin was stronger. • Natural products typically supplied on request by patients.	• “Just like feel it’s like safe, because it’s just like your hormones. It’s not something that, you know. Yeah, that can depress your respiratory system, something like that.”**(P04, 41 M)** • “So, in terms of effectiveness, like I’m not a very pro supplement person. Like if you want to start with supplements, like valerian and stuff by all means, you can try. Maybe it works, like who knows, but there’s not enough research, or there’s not enough, I guess, articles or anything to back me up to say it will work. Therefore, I’m not, super confident or super comfortable recommending it to other people…”**(P09, 30 F)** • “Well melatonin would be safer a bit, as obviously as compared to like benzos because they can have like a greater amount of sedation and everything.”**(P11, 23 F)** • “I think the side effect profile is better for melatonin. Yeah, and then. Like, I feel like a lot of people use them, like doxylamine and Phenergan^⊺^ for less appropriate reasons. Also, doxylamine you can get tolerance so.”**(P06, 22 M)**

^‡^Participant characteristics are indicated in parentheses after each quote. Each participant was assigned a code, with a letter P follow by a number to indicate the interview order, and then their age and gender with M as male and F as female

*Circadin® is the proprietary brand for PR-melatonin 2 mg in Australia

^†^Restavit™ is the proprietary brand for doxylamine 25 mg in Australia

^⊺^Phenergan® is the proprietary brand name for promethazine 10 mg and 25 mg in Australia

**Table 3:** Summary of qualitative thematic synthesis for Theme 2

Theme 2: Preparing for down-scheduling: “learning on the job”		
Subthemes	Summary of synthesis	Illustrative participant quotes ^‡^
Subtheme 2.1. Pharmacists’ perception of melatonin down-scheduling	• Perceived melatonin as a Pharmacist Only Medicine was appropriate balance of public access and safety. • Current pack sizes misaligned with recommended treatment interval.	• “…whenever someone have insomnia, they should talk to a health professional. So if they downgrade to S3 is okay, but I don’t recommend the government to downgrade that to S2 or even non-scheduled.”**(P12, 28 M)** • “Whereas with melatonin, we are seeing an increased need of melatonin. But I think that’s just because it is S3 right now. I think it’s got its place.”**(P03, 23 F)** • “…like doxylamine, it’s like 20 tablets in a packet. The max dose that you can take is 2 tablets, so the maximum that you can take or the shortest period of that is 10 days straight. For melatonin, the dosage is actually 1 a day, and you’ve got 30 tablets inside…I think the pack size is a bit too much for my liking for the treatment of short-term insomnia.”**(P10, 28 M)**
Subtheme 2.2 Preparation for down-scheduling	• Initial preparation focused on compliance with legislative requirements (i.e., age restriction, labelling, placement within pharmacy). • Resources accessed perceived as insufficient, highlighting the need for further targeted training in the clinical applications for melatonin.	• “It’s more in terms of how policies before you supply the medications. Not too much on like educating pharmacists on the medication in terms of like the evidence and the studies.”**(P14, 23 F)** • “…I would welcome much, much more information available for patients, like leaflets, pamphlets, anything that we can hand out to patients that can help them understand a little bit more about [melatonin].”**(P16, 44 M)** • “…I don’t even recall what I learned from those modules. You need like a continuous sort of educational input from someone. It’s not as simple as just, ok here’s your module do it, here’s your CPD points, and off you go recommend [melatonin]” (**P07, 26 F**) • “So, through the PSA^†,^they do mention it a little bit, but didn’t say like what you need to do when you hand out medication. Didn’t say like specific details. They just like an advertisement type, like it down schedule and the what the age, like age limitation they can have.”**(P20, 28 F)**
Subtheme 2.3 Knowledge gaps and training needs	• Participants expressed uncertainty towards melatonin use across age groups. • Key areas identified for up-skilling included dose-response relationships, and guidance for dose titration and use in broader age groups.	• “…with melatonin when do you go from 2[mg] to 5[mg]. Is it just because 2 is not working, or do we start straight on 5? Is there anybody that will be eligible to start straight on 5, you know…There will have to have some sort of guideline.” (**P09, 30 F**) • “Well, I want to see exactly like, how we’re supposed to counsel, how long they’re supposed to use it for, like through S3 like without prescription. Like I don’t even know when to refer them to see a doctor…”**(P08, 23F)** • “…if [melatonin] is solely for those people with low levels of melatonin, we should ask them about their melatonin level instead of just giving it out…we don’t actually really ask about their level of melatonin, we just give it out instead of like asking [if] they really have low levels of melatonin.” (**P21, 24 F**)

^‡^Participant characteristics are indicated in parentheses after each quote. Each participant was assigned a code, with a letter P follow by a number to indicate the interview order, and then their age and gender with M as male and F as female

^†^PSA: Pharmaceutical Society of Australia

**Table 4:** Summary of qualitative thematic synthesis for Theme 3

Impact of down-scheduling: “business as usual”		
Subthemes	Summary of synthesis	Illustrative participant quotes ^‡^
Subtheme 3.1 Patients responding to melatonin down-scheduling	• Public perceived to be misinformed about eligibility criteria. • Majority of direct product requests came from patients < 55 years of age. • Need for educating patients about the risk-benefit of melatonin use.	• “There’s been a lot of misinformation being spread by news outlets and doctors, so a lot of younger patients were under the impression that melatonin was available to them without a prescription.”**(P02, 24 F)** • “Probably the biggest challenge is trying to justify why there is that age limit of 55 years and up in order to receive it. I get it a lot. …So maybe there needs to be more patient education around what Circadin®^*^actually does and why it’s appropriate for people”**(P03, 23F)** • “But everyone like in the late 30s, early 40s coming in asking for it. I think the age limit, the caveat, it’s like a very fine print at the bottom, so it’s not obvious.”**(P17, 30 M)** • “Because a lot of people who previously were getting scripts for it, now you know the doctor’s just said: ‘oh you can just buy it over the counter’”**(P18, 45 M)**
Subtheme 3.2 Regulating melatonin use and being in the firing line	• Pharmacists placed in a difficult position to educate patients and deny melatonin supply. • Eligibility criteria difficult to enforce in practice. • Challenges stem from difficulty monitoring patient and variability in protocol and enforcement between pharmacies. • Relying on patient honesty but many circumvent eligibility criteria.	• “It’s just when that demand gets created through marketing, and it’s not always appropriate demand. I think that’s challenging for pharmacists. It puts people in a difficult ethical position sometimes as well.”**(P05, 51 M)** • “What really surprises me is those that come in and say, ‘well I’ve been able to get it from pharmacy across the road,‘ which is really frustrating because you’re trying to do the right thing.”**(P22, 43 F)** • “I’m sure some people come to us now and just say it’s for their mum or something. I’ve been sus about some of the requests, but I guess we can’t really do anything if that’s what they say…”**(P01, 27F)** • “…if they don’t have history of it, then sometimes some pharmacists are hesitant to hand it out because they don’t know if the patient actually needs it…I think as long as the patient has a history of taking melatonin, then it’s okay to hand it out.”**(P11, 23 F)**
Subtheme 3.3 Managing insomnia: new drug, same approach	• Little to no change observed in management of insomnia. • Down-scheduling of melatonin did not lead to noticeable changes to the sale of OTC/prescribed sleep aids. • Changes observed in prescribing patterns largely attributed to the introduction of Safe Scripts rather than the availability of melatonin.	• “I don’t think the actual sales for other OTC, or even S4 sedatives, have dropped. Some patients are really adamant that melatonin isn’t strong enough for them. They say they would prefer continuing to use what they’re used to.” **(P02, 24 F)** • “The melatonin is a lot more expensive than the Restavit™† as well. So it’s really hard if you do think it’s effective, it’s really hard trying to convince them to change from Restavit™ to melatonin if Restavit™ is so effective in a way that it works straight away.”**(P07, 26 F)** • “Thank God for SafeScripts, without that, we would be like handing it out like a lot…I see it more decrease in benzos because of Safe Scripts, rather than melatonin down-scheduling.”**(P10, 28 M)** • “I’m not too sure about that, but then I can still see a lot of like benzos prescription coming in, even after Circadin® being down scheduled.”**(P19, 25F)**

^‡^Participant characteristics are indicated in parentheses after each quote. Each participant was assigned a code, with a letter P follow by a number to indicate the interview order, and then their age and gender with M as male and F as female

*Circadin® is the proprietary brand for PR-melatonin 2 mg in Australia

^†^Restavit™ is the proprietary brand for doxylamine 25 mg in Australia

#### Theme 1: Role of melatonin: beliefs and attitudes

##### Sub-theme 1.1: Role of melatonin: regulating rather than inducing sleep

Participants described melatonin as a hormone that the body produces to regulate the circadian rhythm. Specifically, for the sleep-wake cycle, melatonin “*tells the body it’s time to go to bed*” (P09, 30 F). Pharmacists/intern pharmacists described the mechanism by which exogenous melatonin worked as *“topping up natural levels”* (P24, 51 F) to regulate sleep rather than inducing sleep. Participants also attributed the onset of insomnia in older adults to age-related declines in melatonin levels. Beliefs about melatonin informed participants’ perception of its role in managing insomnia. Pharmacists/ intern pharmacists perceived melatonin’s onset of effects to be gradual, describing its main function as “*promot[ing] a good sleeping cycle*” (P17, 30 M), which would be suitable for chronic insomnia to recalibrate sleep. In contrast, melatonin played a smaller role in acute insomnia, noting that patients typically wanted quick symptom relief afforded by the fast onset of action from sedating antihistamines.

##### Subtheme 1.2: Comparing sleep aids

Melatonin was considered a separate category of sleep aids, perceived as less pharmacologically active than existing OTC and prescribed sleep aids. However, this reduced activity contributed to a more favourable safety profile for high-risk patient groups (i.e., elderly who are at increased risk of falls). Specifically, pharmacists highlighted the lower risk of residual sedation and that “*you [can’t] really get tolerance with melatonin, because it’s endogenous”* (P06, 22 M). Participants also referred to many instances of observing escalating doses and inappropriate long-term use of sedating antihistamines and/or benzodiazepines in practice. This further reinforced participants’ views that melatonin would be less susceptible to abuse. Although melatonin was often regarded as “*natural*” (P15, 23 F), participants distinguished between proprietary melatonin and herbal sleep aids (e.g., valerian). Their responses highlighted ongoing concerns for herbal products, noting the inconclusive evidence base and variations between a large product base where “*there are so many formulations*” and being uncertain about “*the ideal strength*” (P05, 51 M). Taken together, pharmacists felt less confident about recommending complementary products unless the patient directly requested it.

#### Theme 2: Preparing for down-scheduling: “learning on the job”

##### Subtheme 2.1: Pharmacists’ perception of melatonin down-scheduling

In general, pharmacists/intern pharmacists responded positively to the down-scheduling of melatonin. S3 was perceived as an appropriate balance between public access and mitigating risks associated with self-treatment. While melatonin was considered relatively safe, pharmacists strongly believed their involvement was necessary to establish therapeutic appropriateness and deter patients from self-medicating without supervision from a healthcare professional. In contrast, only one participant (P10, 28 M) felt that melatonin should remain a prescription-only item. This participant was concerned about the larger pack size (i.e., 30 tablets) misrepresenting the recommended short-term 3-week treatment when melatonin is supplied by a pharmacist without a prescription. [41, 42] Further, this participant also noted that a longer-than-intended duration of treatment could potentially delay other more appropriate care by a doctor.

##### Subtheme 2.2: Preparation for down-scheduling

Participants recalled little preparation in response to the down-scheduling. The level of perceived preparedness varied depending on the participant’s role in the pharmacy. A minority of participants who were pharmacy managers or owners (n = 4) responsible for distributing training felt adequately prepared. In contrast, employee pharmacists/intern pharmacists (n = 14) expressed a mismatch between expectations in training and the support received from the owners of the practice or franchise head office. These participants did not feel adequately supported with clinical information about melatonin during the transition phase, often recalling that they were *“learning on the job”* (P03, 23 F). Information related to down-scheduling provided by owners or head office emphasized compliance with legislative requirements such as appropriate labelling, stock placement, and restricting access to customers aged 55 and over. The responsibility for clinical up-skilling fell back on the practicing pharmacist.

Participants reported accessing a range of resources to enhance their knowledge, including educational modules created by the Pharmaceutical Society Australia and the Pharmacy Guild of Australia, articles published in professional journals, slides used during university training, reading the Consumer Medicines Information, and accessing the Australian Medicines Handbook. Although targeted training modules were designed to assist pharmacists with down-scheduling, few participants were aware of their existence. Even among participants who completed the modules, content recall was challenging. The perceived lack of time and financial support for continuing education were also noted as key barriers to training.

##### Subtheme 2.3: Knowledge gaps and training needs

Pharmacists felt proficient in meeting legislative requirements to address their immediate practice needs. Counselling for patients focused on drug-specific advice (e.g., swallowing tablets whole), with all participants advising patients to take melatonin 1–2 hours before bedtime. However, their responses highlighted uncertainty about melatonin use across different age groups. While pharmacists recalled clinical encounters of dispensing or compounding melatonin formulations for children prior to down-scheduling, prescription of melatonin was perceived as the physicians’ remit and never actively questioned beyond “*doctor said it was fine*” (P07, 26F). The down-scheduling has prompted pharmacists’/intern pharmacists’ interest in understanding the dose-response relationship between melatonin and sleep promotion, the circadian science underpinning the effects of melatonin, and thresholds for melatonin levels to guide dosage titration. Participants also wanted further information to distinguish the use case between immediate- and sustained-release melatonin formulations.

#### Theme 3: Impact of down-scheduling: “ business as usual “

##### Subtheme 3.1: Patients responding to melatonin down-scheduling

Pharmacists across practices unanimously anticipated high demand in product-specific requests for melatonin, given the widespread publicity campaigns. Patients responded to the advertising campaigns enthusiastically with the number of direct product requests ranging from 2 to 70 each week.

However, pharmacists felt that the publicity campaigns were “*misleading*” (P05, 51 M) and oversimplified the process for accessing S3 medicines, which contributed to the public’s confusion about their eligibility to access melatonin without a prescription. In fact, a small subset of pharmacists (n = 3) recalled patients being advised by their doctor to approach their pharmacist about melatonin despite being under 55 years of age. As a result, during the initial phase of the down-scheduling, nearly all requests came from adult patients outside of the eligible age group (i.e., under 55 years of age) with the expectation that melatonin would be supplied.

##### Subtheme 3.2: Regulating melatonin use and being in the firing line

While the guidelines appeared straightforward, patient misinformation about eligibility placed pharmacists/intern pharmacists in the difficult position of educating patients and denying supply. Altercations that arose when denying the supply of melatonin were a recurring theme among participants during the initial transition period of down-scheduling. One participant reported supplying PR-melatonin in younger adults to avoid patient conflict since they didn’t *“think there’s enough side effects for it (melatonin) to be an issue*” (P15, 23 F).

Melatonin supply procedures also varied between participants, ranging from entering data into dispensing software for each supply, asking for proof of identity to verify age, to simply relying on patient honesty when reporting their age. The lack of consistency between pharmacies and the extent to which eligibility was applied further contributed to pharmacist-patient tensions where “*it gets really ugly.*” (P07, 26 F). Since the initial down-scheduling, patients appeared to have circumvented the eligibility criteria by lying about their age, requesting supplies for older family members, or going to different pharmacies. The current supply process also made tracking melatonin use difficult, with one participant suggesting that melatonin could perhaps “*be S3R*^4^*“* (P22, 43 F); [43] that is, supplies are recorded as if they were a Schedule 4 prescription and entered into an electronic database that provides supply history for a patient. However, in practice, the recommended 3-week treatment duration was rarely enforced as pharmacists were unaware or reassured of therapeutic need based on patients’ history of melatonin use.

##### Subtheme 3.3: Managing insomnia: new drug, same approach

The addition of PR-melatonin did not appear to change pharmacists’ approach to managing insomnia. Most participants reported little to no change in the sale of other OTC sleep aids or the frequency of patient requests. For the small subset of pharmacists (n = 2) observing a reduction in benzodiazepine prescribing, this was largely attributed to the introduction of SafeScript [44], a real-time prescription monitoring system, rather than an increased awareness of melatonin. Despite the down-scheduling, pharmacists rarely recommended melatonin for managing insomnia in criteria-concordant patients unless it was for symptom-based requests (n = 10). However, such requests were rare due to the widespread publicity and marketing associated with the down-scheduling, with most patients making direct product requests for melatonin. Interestingly, patients already taking another OTC sleep aid (e.g., doxylamine) were resistant to change to melatonin. Factors contributing to the resistance included the high costs of melatonin relative to other OTC sleep aids and patients not wanting to discontinue an effective product as “*… melatonin isn’t strong enough…”* (P02, 24 F).

## Discussion

In this qualitative study we explored Australian community pharmacists’ experiences, views and attitudes towards the down-scheduling of melatonin. Our findings highlight several challenges when enforcing eligibility criteria in practice including variation in practice between pharmacies, misalignment between pack sizes and treatment duration and public misinformation  about the eligibility for accessing melatonin without prescription. Pharmacists/intern pharmacists further articulated training needs to address knowledge gaps in sleep and circadian health. Table 5 outlines the research and practice implications of the findings.

**Table 5:** Practice and research implications

Issues identified from pharmacists’ perspective	Recommendations
Difficulty enforcing age-related restrictions for over-the-counter supply of melatonin	Developing resources that clarifies clinical rationale underpinning age-related use of melatonin.
Difficulty tracking the supply	Standardising the recording of over-the-counter supply of melatonin.
Pack sizes do not reflect the recommended treatment duration	Smaller pack sizes to better reflect the recommended duration of therapy.
Variations in practice between pharmacists and pharmacies	Standardizing guidelines and protocols to unify practice, reducing variability in experience for the patient.
Transition phase focused on regulatory compliance over clinical evidence	Development of education resources to support pharmacists’ expanding scope of practice in sleep health.
Misinformed patient expectations about melatonin and over-estimating its safety	Public health education bringing greater awareness about the risks and benefits associated with melatonin use and regulatory compliance.

While participants supported the down-scheduling of melatonin, our findings highlighted a disconnect between practice and guidelines for the S3 supply of PR-melatonin in community pharmacy. In practice, participants found it challenging to reconcile the lack of objective measures for gauging the age-related decline in melatonin levels, rendering enforcement of the age restriction challenging, which often caused patient-pharmacist tension. The uncertainty around the age-related use of melatonin is also reflected in the survey where the majority (n = 19) did not feel comfortable broadening the age range of melatonin use as an over-the-counter medication (Fig. 1). Further, pack sizes of PR-melatonin (30 tablets) implied a 4-week treatment period, while current guidelines recommend acute treatment for 3-weeks [42, 45]. Even targeted training resources designed to upskill pharmacists endorsed the interpretation of “short-term” to include up to 13 weeks of treatment for practical purposes [18], leading to inconsistencies in the counselling of patients. In fact, participants in our study drew on additional proxy criteria in their decision-making, conflating therapeutic appropriateness with prior history of prescribed melatonin use [46, 47]. Under these circumstances, uncertainty in pharmacists’ clinical decision-making is likely to arise, leading to deviation from guidelines and variability in practice between pharmacists and pharmacies [48], resulting in longer-than-intended melatonin use and delaying other, potentially more appropriate, medical care [49]. Such practice experiences may also explain why the majority of participants (n = 21) expressed interest in engaging in further continuing professional development activities related to melatonin and sleep (Fig. 1). Taken together, there is a need to develop practice resources that provide greater clarity on the clinical rationale underpinning the age restriction in the current guidelines, while refining and unifying supply protocols and amending pack sizes to reflect pharmacists’ practice needs.

For the majority of participants (n = 20), the down-scheduling of melatonin did not change the sales of other over-the-counter sleep aids (OTC) (Table 1). This may in part relate to the fact that not all pharmacists (n = 9) believed that melatonin was a better alternative to existing OTC sleep aids since healthcare professional beliefs influence their clinical decisions [50]. However, addressing misinformed patient expectations was perceived as most challenging by pharmacists. Patients were unaware of the eligibility criteria for accessing melatonin and perceived the age restriction as nonsensical. These views may stem from public beliefs around ‘naturalness’ and presumed safety of melatonin [51, 52], coupled with the lack of public awareness around the conditions entailing the S3 supply of melatonin from a pharmacist [53]. In this context, patients may underestimate the risks associated with melatonin use [54], thus influencing them to circumvent the eligibility criteria to access melatonin, as observed by our participants [55]. This has important safety implications; the physiological effects of melatonin extend beyond sleep and may affect the endocrine, reproductive, cardiovascular, immunologic, and metabolic systems, particularly among patients with comorbidities [56, 57]. For instance, melatonin use has been associated with impaired glucose tolerance [58], and interference with immunosuppressive therapies [59]. Indirectly, unauthorised access by a broader patient population (i.e., those < 55 years of age) can increase the risk of unintentional paediatric exposure to melatonin. In the United States, where melatonin access is unregulated, there has been a rapid rise over the last decade in paediatric hospitalisations and severe outcomes due to unintentional ingestions by children under the age of 5 [60]. While the initial patient-pharmacist tension may have subsided, educating the public, and raising awareness about the potential safety concerns of melatonin remains an important public health issue.

Participants endorsed a good understanding of how melatonin works in the body and were confident in counselling patients about melatonin use. However, survey responses showed that counselling mainly focused on drug-specific advice (e.g., swallowing tablets whole), with administration 1–2 h before bedtime being the most prevalent (Fig. 2). However, this approach does not account for patients’ highly variable circadian timing and can lead to patients’ circadian timing shifting in the wrong direction, further worsening the sleep complaint [61]. In fact, pharmacists’ description of melatonin’s mechanism of action reflects a gap in understanding of its chronobiotic potential in modulating circadian timing with only 10 participants describing its main function in sleep as a “chronobiotic agent” (Table 1). This has important practice implications in light of the proposed plans by the TGA to expand the indication of OTC melatonin for mitigating jetlag in younger adults [45]. In the context of jetlag, administration of treatment requires consideration of the individual’s circadian timing relative to the direction of travel (i.e., melatonin taken at night for eastward travel to phase advance) [62]. As such, expanding sleep health education to include principles of chronotherapy and circadian science represents an important area of development within the pharmacy curriculum and ongoing continuing professional development [30].

## Strengths and limitations

A key strength of this study was the in-depth exploration of the practice experiences among practising community pharmacists/ intern pharmacists, providing a comprehensive snapshot of the current state of practice. The study design adhered to COREQ checklist [40]; and qualitative data abstraction had a high degree of intercoder reliability (Kappa score = 0.89).

However, there are some limitations to consider. Our participants were a self-selected sample who may be more interested in melatonin and sleep health than the community pharmacists more generally and thus may have different training needs and interests. Participants were primarily female, limiting the views of male pharmacists but the gender split in our study is nonetheless representative of the current gender distribution in practising pharmacists [27]. Relatedly, recruited pharmacists practised in metropolitan NSW, and thus views may not necessarily transfer to other settings, particularly those in regional/remote communities. Nevertheless, NSW is the most populous state in Australia and therefore represents the largest population of practising pharmacists, where participants’ perspectives provide  valuable insight for informing and guiding the refinement of practice guidelines. Future studies should consider a broader sampling frame to include pharmacists across all states and territories of Australia, but particularly regional/remote communities. In addition, ratings of participants’ knowledge, beliefs, and attitudes towards melatonin for sleep were not formally validated, warranting further research and development as pharmacists become increasingly involved in the delivery of sleep health services. Recall bias also needs to be considered as interviews were conducted approximately a year after the down-scheduling of melatonin. Current findings may reflect a more stable integration of practice compared to initial period of transition, but nonetheless, provides insight for understanding how pharmacists navigated this regulatory change. Transcripts were not checked by participants as this task would not be feasible given time constraints of the practice environment. Participants who indicated their interest were noted and will be sent feedback upon completion of the study.

## Conclusion

In conclusion, pharmacists were in favour of down-scheduling of melatonin but did note several challenges in the transition phase. Despite pharmacists meeting immediate practice needs, further training in the area was warranted. Many felt a disconnect between current supply protocols and practice, contributing to patient-pharmacist tensions when supply is denied. Findings further allude to the need to better educate the public about the risk-benefit profile of melatonin, while refining and unifying current supply protocols and amending pack-sizes to reflect the recommended short-course treatment.

### Electronic supplementary material

Below is the link to the electronic supplementary material.

Supplementary Material 1

Supplementary Material 2

Supplementary Material 3

Supplementary Material 4

## Footnotes

1 Schedule 3 (S3) Medicines are defined in the Poisons Standard and allows for certain therapeutic agents available to the public without a prescription through pharmacist advice. 15.Poisons Standard October 2022 (SUSMP No. 37) (Cth), (2022).

2 Inappropriate timing of exogenous melatonin may lead to the body’s natural cycle and the external 24-hour environment to desynchronise. 19.Andrews CD, Foster RG, Alexander I, Vasudevan S, Downes SM, Heneghan C, et al. Sleep–Wake Disturbance Related to Ocular Disease: A Systematic Review of Phase-Shifting Pharmaceutical Therapies. Transl Vis Sci Technol. 2019;8(3):49-.

3 Phase advance moves bedtime and wake-up times to earlier in the day and may be socially incompatible.

4 Schedule 3 Recordable.
