# Supplementary material for: Pharmacists’ perspectives and attitudes towards the 2021 down-scheduling of melatonin in Australia using the Theoretical Domains Framework: a mixed-methods study
Source: Int J Clin Pharm. 2023 Jun 24;45(5):1153–66. doi: 10.1007/s11096-023-01605-w (PMC10600292; doi:10.1007/s11096-023-01605-w)
Supplement: Supplementary file 1 — Supplementary Material 1 [file 11096_2023_1605_MOESM1_ESM.docx]

Community Pharmacists’ Provision of Melatonin Formulations – A Practice Survey

**Part 1: Demographic Information**

| 1 | Please note that this survey is designed for registered and intern pharmacists whose principal place of practice is in an Australian community pharmacy. If you agree with this statement and the Participant Information Statement, please click continue.   1. Continue 🡪 *continue to Question 2* 2. Exit 🡪 *end survey* |
| --- | --- |
| 2 | What is your current role at your principal place of practice?   1. Registered pharmacist 2. Intern pharmacist 3. Pharmacist manager 4. Pharmacist in Charge 5. HMR accredited pharmacist 6. Locum owner 7. Locum pharmacist |
| 3 | What is your age? __________ years |
| 4 | Which gender do you identify as?   1. Male 2. Female 3. Other |
| 5 | How many years have you practiced as a pharmacist or an intern? __________ years |
| 6 | In what location setting is your principal place of practice situated?   1. Metropolitan 2. Regional 3. Rural 4. Remote 5. Other; please specify __________ |
| 7 | What is the postcode of the town or region where your principal place of practice is located?  ____ |
| 8 | What type of pharmacy is your principal place of practice?   1. Independent or private pharmacy 2. Franchise pharmacy 3. GP clinic pharmacy 4. Hospital-based pharmacy 5. Other; please specify __________ |
| 9 | Please select any additional professional services your principal place of practice offers.   1. Dosage administration aids e.g.: Webster paks, sachets 2. Accredited compounding or aseptic compounding services e.g.: PCCA, Medisca 3. MedsCheck and/or Diabetes MedsCheck 4. Community opioid replacement program (OTP) 5. Clozapine clinic e.g.: ClopineCentral, Clopine Connect 6. Health screening and/or promotion services; please specify the type of service provided __________ 7. Vaccination clinic – including COVID-19 vaccination/booster rollout 8. Home medicines review (HMR) 9. Other; please specify __________ |
| 10 | On average, what is the number of prescriptions dispensed PER DAY at your principal place of practice? __________ scripts |
| 11 | On average, how many patients or customers do you see with sleep complaints at your principal place of practice? __________ |

**Part 2: Current State of Practice – Provision of Melatonin in Community Pharmacy**

| 12 | Does your principal place of practice provide compounded melatonin formulations?   1. Yes 2. No |
| --- | --- |
| 13 | Does your principal place of practice sell other melatonin-containing products in the vitamins, supplements or herbal medicines section of the pharmacy?   1. Yes 2. No |
| 14 | On average, how many direct requests or enquiries for melatonin products do you receive each week at your principal place of practice? __________ |
| 15 | Which melatonin product is most commonly requested by patients at your principal place of practice? __________ |
| 16 | Since the down-scheduling of Circadin/Melotin (prolonged-release melatonin tablet) in 1^st^ June 2021, the number of enquiries around melatonin and melatonin-containing products have:   1. Increased 2. Decreased 3. Stayed the same |
| 17 | Since the down-scheduling of Circadin/Melotin (prolonged-release melatonin tablet) in 1^st^ June 2021, the sale of other Schedule 3 sleep aid products such as doxylamine has:   1. Increased 2. Decreased 3. Stayed the same |
| 18 | Has your principal place of practice introduced new clinical processes or protocols to accommodate for the down-scheduling of prolonged-release melatonin?   1. Yes 🡪 *go to Question 19* 2. No 🡪 *go to Question 20* |
| 19 | Please describe the processes or protocols in place to mediate provision of Schedule 3 prolonged-release melatonin.  ______________________________________________________________________________________________________________________________________________________ |
| 20 | Do you record the supply of Schedule 3 prolonged-release melatonin on your dispensing software?   1. Yes 2. No |
| 21 | Since the down-scheduling of Circadin/Melotin (prolonged-release melatonin tablet) in 1^st^ June 2021, the challenges or disruption to workflow due to Circadin/Melotin requests have:   1. Increased, more than usual 2. Decreased, less than usual 3. Remained unchanged 4. Other; please specify __________ |
| 22 | Which one of the following have been the most common reason for direct product requests of Schedule 3 prolonged-release melatonin at your principal place of practice?   1. Insomnia in patients aged 55 years and over 2. Insomnia in an otherwise healthy adult under age of 55 years 3. Jet lag in an otherwise healthy adult 4. Sleep disturbance in children under 18 years of age 5. Other; please specify __________ |

**Part 3: Pharmacists’ Understanding of Melatonin in Sleep Medicine**

| 23 | What do you consider to be the main function of melatonin in sleep modulation?   1. Sleep-inducing agent 2. Chronobiotic agent 3. Hormonal agent 4. All of the above 5. Other; please specify __________ |
| --- | --- |
| 24 | What are the key counselling points you go through with your patient when providing scheduled melatonin products?   1. Take 1-2 hours before intended bedtime (Circadin/Melotin); give 30-60 minutes before intended bedtime (Slenyto) 2. Circadin/Melotin: this medication may affect your ability to drive, and may interact with alcohol 3. Do not crush, or chew, the tablet 4. Sleep hygiene counselling 5. Non-pharmacological strategies to aid sleep 6. Provision of patient-friendly information resources e.g.: Sleep Health Foundation 7. Other; please specify __________ |

**Part 4: Beliefs and Attitudes towards Melatonin and Sleep Health**

| *For the following statements, please rate the extent to which you agree or disagree with the following statements. Keep in mind that there is no right or wrong answer to these questions.* | |
| --- | --- |
| 25 | I have a good understanding of how melatonin works on the body.   1. Strongly agree 2. Agree 3. Neutral 4. Disagree 5. Strongly disagree |
| 26 | In general, melatonin is a better alternative to other over-the-counter sleep aids such as doxylamine.   1. Strongly agree 2. Agree 3. Neutral 4. Disagree 5. Strongly disagree |
| 27 | In general, melatonin is safer in comparison to other prescription medicines (e.g.: benzodiazepines, z-drugs) used for insomnia.   1. Strongly agree 2. Agree 3. Neutral 4. Disagree 5. Strongly disagree |
| 28 | I received adequate training around the supply of melatonin products in a pharmacy setting, including Schedule 3 modified-release melatonin.   1. Strongly agree 2. Agree 3. Neutral 4. Disagree 5. Strongly disagree |
| 29 | I recognise that insomnia is a common comorbidity seen in patients with certain health conditions, such as Attention Deficit Hyperactive Disorder (ADHD) and chronic pain.   1. Strongly agree 2. Agree 3. Neutral 4. Disagree 5. Strongly disagree |
| 30 | I am confident in counselling patients, carers, or parents about the use of melatonin products (including Schedule 3 modified-release melatonin) in my practice.   1. Strongly agree 2. Agree 3. Neutral 4. Disagree 5. Strongly disagree |
| 31 | I am comfortable with patients of all ages being able to access melatonin products, including Schedule 3 modified-release melatonin, without a prescription.   1. Strongly agree 2. Agree 3. Neutral 4. Disagree 5. Strongly disagree |
| 32 | Sleep health is an important concern for patients, carers, or parents I see in my practice.   1. Strongly agree 2. Agree 3. Neutral 4. Disagree 5. Strongly disagree |
| 33 | Sleep health is a major concern I see for patients, carers, and parents with comorbid health conditions such as Attention Deficit Hyperactive Disorder (ADHD) and chronic pain.   1. Strongly agree 2. Agree 3. Neutral 4. Disagree 5. Strongly disagree |
| 34 | I am interested in participating in professional development activities that are related to the effects of melatonin on sleep.   1. Strongly agree 2. Agree 3. Neutral 4. Disagree 5. Strongly disagree |

**Part 5: Recommending Healthy Sleep Habits**

| *During a typical week in your practice, please rate the frequency you provide the following advice or care for your patients, carers, or parents to improve sleep health.* | |
| --- | --- |
| 35 | I educate patients, carers, or parents about avoiding caffeine and alcohol intake close to bedtime.   1. Always 2. Often 3. Sometimes 4. Rarely 5. Never |
| 36 | I educate patients, carers, or parents about relaxation techniques to unwind before bedtime.   1. Always 2. Often 3. Sometimes 4. Rarely 5. Never |
| 37 | I educate patients, carers, or parents about maintaining a good sleep environment e.g.: keeping the room dark, optimising temperature, minimising noise, etc.   1. Always 2. Often 3. Sometimes 4. Rarely 5. Never |
| 38 | I educate patients, carers, or parents about the importance of maintaining a regular sleep-wake schedule.   1. Always 2. Often 3. Sometimes 4. Rarely 5. Never |
| 39 | I educate patients, carers, or parents about options for tracking sleep either digitally (e.g.: Apple Watch, Fitbit) or manually (e.g.: using a sleep diary or habit tracker).   1. Always 2. Often 3. Sometimes 4. Rarely 5. Never |
| 40 | I educate patients, carers, or parents about the potential risks and harms of long-term medication use for insomnia.   1. Always 2. Often 3. Sometimes 4. Rarely 5. Never |
| 41 | I screen patients for sleep disorders like insomnia and obstructive sleep apnoea.   1. Always 2. Often 3. Sometimes 4. Rarely 5. Never |
| 42 | I screen patients with comorbidities such as neuropsychiatric or respiratory disorders, for possible co-existing sleep disorders like insomnia and obstructive sleep apnoea.   1. Always 2. Often 3. Sometimes 4. Rarely 5. Never |
| 43 | I refer patients, carers, and parents to see their doctor to get further help for their sleep concerns.   1. Always 2. Often 3. Sometimes 4. Rarely 5. Never |
| 44 | I refer patients to see a psychologist to receive cognitive behavioural therapy for insomnia.   1. Always 2. Often 3. Sometimes 4. Rarely 5. Never |

**Part 6: Comments**

| 45 | Is there anything else about melatonin use in community pharmacy that you would like to share with us?  ______________________________________________________________________________________________________________________________________________________ |
| --- | --- |
| 46 | Would you like to be interviewed by one of our researchers about melatonin use in community pharmacy?   1. Yes 🡪 *go to Question 47* 2. No 🡪 *go to Question 48* |
| 47 | Please provide us with your contact details and one of our researchers will contact you to organise an interview with you at a suitable time.   1. Name: __________ 2. Email: __________ 3. Phone number: __________ |
| 48 | Would you like to receive feedback about the overall results of this study?   1. Yes 🡪 *go to Question 49* 2. No 🡪 *end survey* |
| 49 | Please provide your preferred method of correspondence for the research team to provide you with feedback at the conclusion of this study.   1. Email: __________ 2. Postal address: __________ |

Thank you for participating in our research study!
