# Supplementary material for: Pharmacists’ perspectives and attitudes towards the 2021 down-scheduling of melatonin in Australia using the Theoretical Domains Framework: a mixed-methods study
Source: Int J Clin Pharm. 2023 Jun 24;45(5):1153–66. doi: 10.1007/s11096-023-01605-w (PMC10600292; doi:10.1007/s11096-023-01605-w)
Supplement: Supplementary file 2 — Supplementary Material 2 [file 11096_2023_1605_MOESM2_ESM.docx]

**Online Resource 2: Interview guide**

**Exploring the Supply of Melatonin Products in Community Pharmacy – Interview Guide**

Thank you for agreeing to taking part in our study. I would like to remind you that participation in this research is voluntary, and you may withdraw your participation at any time. We are recording this interview so that your thoughts can be accurately captured for analysis. If you prefer, you can switch off your camera. At any point during the interview, if you feel uncomfortable, please let me know and I can stop the recording. Can we go ahead with the interview?

|  | **Question** | **Prompts** | **Rationale** |
| --- | --- | --- | --- |
| 1 | What do you see is the role of melatonin in sleep?  What is your opinion on the use of melatonin for sleep, when compared to other products available over the counter e.g.: doxylamine, promethazine, valerian? | - Classification i.e.: chronobiotic vs. sleep aid - Perceived mechanism of action - Counselling points, and whether it varies depending on the patient e.g.: existence of comorbid conditions such as Attention Deficit Hyperactive Disorder or chronic pain - Concerns about adverse effects (short- and long-term) | To understand the pharmacists' beliefs around melatonin use in sleep, and how it affects the sleep-wake cycle. |
| 2 | What is your opinion on the current availability of melatonin products in Australia? | - Types of products seen in practice e.g.: homeopathic, supplemental, proprietary, compounded - Diversity of products available in the current market - How are patients procuring melatonin products e.g.: through direct requests at the pharmacy, online, etc. - Cost of melatonin products available in Australia - Safety and quality assurance of products sourced online | To understand the pharmacists' perspectives around availability of melatonin products in Australia and its implications in practice. |
| 3 | The most recent regulatory change around melatonin products in Australia was the down-scheduling of prolonged-release melatonin (*Circadin/Melotin*) in June 2021. What were your initial thoughts when you heard about the down-scheduling? | - Positive or negative reaction - Implications in current practice - Confidence around counselling and educating other pharmacy staff about over-the-counter melatonin | To gain insight into the attitudes pharmacists have around the recent down-scheduling of melatonin. |
| 4 | How did your pharmacy prepare for the down-scheduling of prolonged-release melatonin (*Circadin/Melotin*) in June 2021? | - Was there adequate time given for the pharmacy to prepare and adapt to the change? - Do you think the way information was conveyed to pharmacists and pharmacies around regulatory change could have been improved? - What were some of the challenges that you have encountered since the down-scheduling? |  |
| 5 | Does your pharmacy have any protocols being followed to facilitate Schedule 3 supply of prolonged-release melatonin?   - If yes; please describe the processes involved. - If no; do you plan to use one in the future? | - Recording patient information on the dispensing software - Any additional measures employed to mitigate supply - Loopholes in the current system? - Counselling: dose, duration of use, sleep hygiene advice | To understand processes and additional measures employed in Schedule 3 provision of prolonged-release melatonin. |
| 6 | How have patients in your pharmacy responded to the over-the-counter availability of a proprietary melatonin product? | - Have you seen any trends in the product requests you have received i.e.: increases in request for melatonin? - Have you seen any changes in product sales when compared to:   - Other over-the-counter sleep aids e.g.: doxylamine, promethazine   - Prescription sedative hypnotics e.g.: benzodiazepines - Have you had any questions or confusions expressed by patients in the differences between different products? - Have you had any challenges around enquiries received for patients under 55 years of age? | To understand the impact of regulatory changes on practice, and patients encountered in the pharmacy. |
| 7 | I also want to prompt around the response received by parents or caregivers of children, who are also large target market for melatonin use.    Have there been enquiries received regarding access of over-the-counter melatonin for children? How have you responded to these questions? | - How have you seen parents or caregivers access melatonin for their child in the past e.g.: compounding, online, etc.? - Have you seen children under 18 years of age use Circadin or Melotin via a prescription? What has been your general advice for parents and caregivers prescribed Circadin or Melotin for their child e.g.: crushing the tablet or adding into yoghurt/juice? - How have you seen parents or caregivers view melatonin from their perspective e.g.: whether it is supplemental vs. medicinal, natural vs. synthetic, safe vs. unsafe, long-term safety concerns? - Has there been an increase of enquiries from parents or caregivers following the down-scheduling in June 2021? - What has been the response around restriction of provision as a patient under 55 years of age? - Have you had conversations around Schedule 4 products available for children i.e.: Slenyto 1mg and 5mg? What has been the uptake of this formulation so far? | To understand the trends in melatonin use within the paediatric population, and the impact of regulatory changes on parents or caregivers of children requiring melatonin use. |
| 8 | Is there anything else that you would like to tell us about the provision of melatonin products in community pharmacy? |  |  |
