# Supplementary material for: Pharmacists’ perspectives and attitudes towards the 2021 down-scheduling of melatonin in Australia using the Theoretical Domains Framework: a mixed-methods study
Source: Int J Clin Pharm. 2023 Jun 24;45(5):1153–66. doi: 10.1007/s11096-023-01605-w (PMC10600292; doi:10.1007/s11096-023-01605-w)
Supplement: Supplementary file 3 — Supplementary Material 3 [file 11096_2023_1605_MOESM3_ESM.docx]

Sub-theme

Thematic Category

Code

Sub-code

Theme

**Online Resource 2a. Coding tree representing the codes, categories, and sub-themes that emerged from interviews for Theme 1**

Sub-theme

Thematic Category

Code

Sub-code

Theme

**Online Resource 2b. Coding tree representing the codes, categories, and sub-themes that emerged from interviews for Theme 2**

Sub-theme

Thematic Category

Code

Sub-code

Theme

**Online Resource 2c. Coding tree representing the codes, categories, and sub-themes that emerged from interviews for Theme 3**
